# Image Preprocessing in Classification and Identification of Diabetic Eye Diseases

**DOI:** 10.1007/s41019-021-00167-z

**Published:** 2021-08-17

**Authors:** Rubina Sarki, Khandakar Ahmed, Hua Wang, Yanchun Zhang, Jiangang Ma, Kate Wang

**Affiliations:** 1 Victoria University, Ballarat Road, Melbourne, VIC 3011 Australia; 2 Federation University, Mount Helen, Australia; 3 RMIT University, Melbourne, Australia

**Keywords:** Diabetic eye disease, Image processing, Convolution neural network

## Abstract

Diabetic eye disease (DED) is a cluster of eye problem that affects diabetic patients. Identifying DED is a crucial activity in retinal fundus images because early diagnosis and treatment can eventually minimize the risk of visual impairment. The retinal fundus image plays a significant role in early DED classification and identification. An accurate diagnostic model’s development using a retinal fundus image depends highly on image quality and quantity. This paper presents a methodical study on the significance of image processing for DED classification. The proposed automated classification framework for DED was achieved in several steps: image quality enhancement, image segmentation (region of interest), image augmentation (geometric transformation), and classification. The optimal results were obtained using traditional image processing methods with a new build convolution neural network (CNN) architecture. The new built CNN combined with the traditional image processing approach presented the best performance with accuracy for DED classification problems. The results of the experiments conducted showed adequate accuracy, specificity, and sensitivity.

## Introduction

**Fig. 1:**
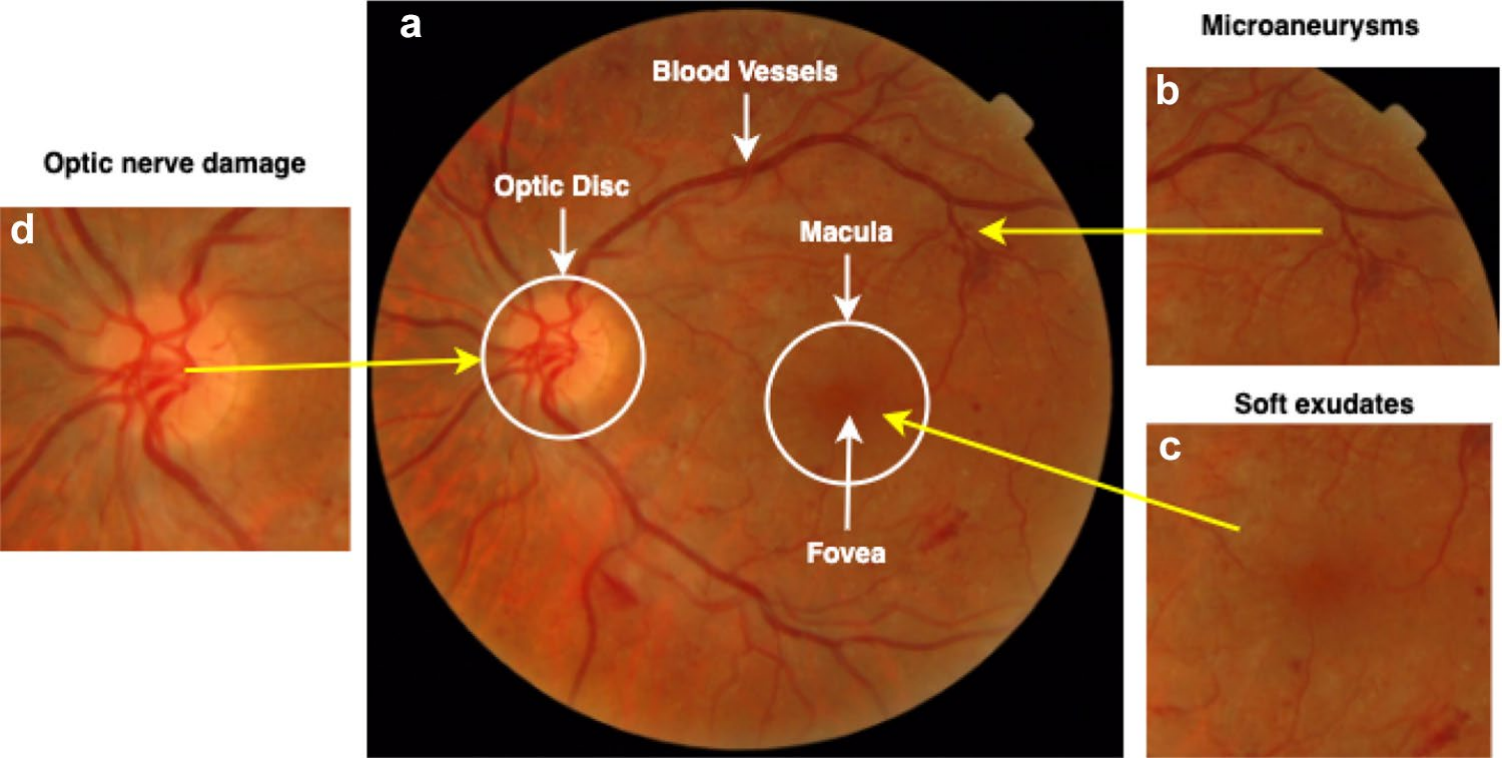
Early DED complication in retina; **a** Anatomical structure of the retina; **b** Microaneurysms- narrow buldges in blood vessels (diabetic retinopathy); **c** Soft exudates in macula (diabetic macular edema); (d) Optic nerve damage (glaucoma); and **e** cataract

Diabetic eye disease is the most common complication in diabetes, in which retinal fundus imaging is the most commonly adopted procedure because of its sensitivity in the diagnosis of DED [21]. The analysis of the severity and intensity of DED correlated with a patient having diabetes is typically attended by Ophthalmologists based on the lesion presents in retinal fundus images [50]. For instances, Fig. 1 presents the details on lesions that need to be identified from retinal images are; (i) Extra growth of blood vessels and damage or rupture in the tiny blood vessels in the retina (microaneurysms), often known as an early stage of diabetic retinopathy (DR); (ii) Built-up fluid causing swelling in the macular region or often form soft exedutes known as diabetic macular edema (DME). The common reason for blindness and vision loss, and (iii) Damage in the optic nerve and blood vessel rupture causes intraocular pressure that damages the optic nerve to inadequate, causing glaucoma (Gl). This eye condition is irreversible. While the number of diabetic patients is exponentially growing, there is also a rise in the number of retinal fundus images obtained by screening campaigns, which, in effect, causes a considerable labor-intensive and time-consuming complexity on the medical experts. This complexity has driven the development of an automated retinal lesions detection system.

Deep learning (DL) in machine learning (ML) has shown a significant impact on different science fields over the last few years [18, 22]. Such as the advancements in image and speech recognition, the ability to train artificial data that beat human players in games like ATARIS [30] and GO [42], and the development of new creative images using methods like Generative Adversarial Networks (GAN) [49], to learn the interaction between review texts and star ratings for prediction [12, 37] and as well as music [7]. Several of these activities were considered onerous to be accomplished by algorithms before the development of deep learning. Deep learning (DL) system plays a significant role in healthcare [11, 39, 48].

In deep learning, a pre-trained CNN network can be used to *transfer learning* from source task to target task with a limited number of images or minimise training time [28, 36]. The most popular *transfer learning* method is to fine-tune the pre-trained network. Regardless of the nature of the training model (pre-trained model or a new model), image data sets are typically preprocessed prior to training CNN architectures in various ways, such as image resizing, image quantity, image standardisation, and image enhancement. Improving the classification performance of the CNN model is limitless research, and the image quality in the data set has a significant impact on the overall performance of the architecture.

### Motivation

Spectacular developments in the fields of artificial intelligence, computer vision and deep learning have led to remarkable results in image classification and vision tasks over the last several years, primarily through the use of DL [27]. However, in medical imaging (e.g., retinal fundus images), an early-stage identification of lesions and abnormalities is still the open issue reported in previous literature by Lam. et al. [27]. In their study they mentioned deep neural networks are struggling to learn enough in-depth features to identify aspects of mild disease; 93 percent of mild cases are wrongly classified as a healthy eye. Therefore this research presents the system in which traditional image processing techniques and the state-of-the-art CNN combined to analyze early-DED disease. This paper articulates a research study using a small volume of the open-source retinal image database for in-depth learning evaluation between normal and mild DED classification.

### Contributions

Therefore, in this research article, the main objective is to achieve the highest accuracy, sensitivity, and specificity than the existing deep learning models. The technique we used in this paper is the combination of traditional image processing methods for image enhancement and segmentation and then train in deep learning algorithms. We explore the significance of traditional image preprocessing in enhancing the early stage DED detection accuracy by using DL models. The advancement of this technology does not indicate the complete substitution of an ophthalmologist. Rather, it allows ophthalmologists more reliably diagnose DED. The contribution of this paper in the diagnosis of early DED can be classified into the following groups;

- Image enhancement: green channel extraction, contrast limited adaptive histogram equalization (CLAHE), and illumination correction was used to enhance the original image;
- Image segmentation: Region of Interest (ROI) such as blood vessels, macular region and optic nerve segmented from retinal fundus images;
- Pre-trained model: high-performance models were selected to classify the processed and segmented retinal fundus images;
- Build a new CNN model and training the model from scratch with processed and segmented retinal fundus images.

## Related Works

Early detection of DED in retinal fundus images relies on a clinical technique to visualize a comprehensive set of features and localization within the image [4, 40]. Detection is challenging for diabetic patients with early DED stages because it depends on the existence of microaneurysms (bulges in blood vessels), fluid leakage from blood vessels, soft exudates formation, and damage of the optic nerve on retinal fundus images. The stages of diabetic eye disease are shown in Fig.1.

In the past, automated DED diagnostics have been explored to ease the burden on ophthalmologists and minimise diagnostic inconsistencies [31]. There are studies, which used lesion-based detection, an author like; Gharaibeh et al. [16] presented a new approach to detect microaneurysms in retinal fundus images. Their work includes preprocessing methods like blood vessel segmentation, fovea localization, and elimination. Then they used a combination of neural networks and fuzzy logical models for feature extraction and classification. Their study addressed the binary classification of diabetic retinopathy into two categories (microaneurysms and non-microaneurysms).

Moreover, a range of several other features than microaneurysms are appropriate for the diagnosis of DED. Similarly, Kaur et al. [25] proposed region-based segmentation and detection of the lesion and then classified using pixel-based classification and determine the severity level of the retinal disease. Karegowda et al. [24] detected exudates in diabetic retinopathy using decision tree and GA-CFS techniques as input to backpropagation neural network. They classified the normal eye and eye with exudate. The results obtained were insufficient to provide reliable classification accuracy and do not result in efficient noise removal. Sopharak et al. [47] presented a fuzzy c-means and clustering-based exudate identification method. Their work mostly relies on the identification of optic disc and the elimination of blood vessels. According to the results obtained, the exudates are identified without their characteristics. Jenuja et al. [23] present a method based on the optic disc and optic cup segmentation. The proposed method uses dual neural networks that operate in combination with the optical cup and disc parts. The aim of this proposed method is to efficiently segment the optic cup and disc of a retinal fundus image. The results of the classification of various stages of glaucoma are not given. Earlier Gulshan et al. [17] and Gargeya et al [15] presented CNN for DR detection using fundus images. They achieved specificity and sensitivity in the range of 90 percent for (normal/mild to moderate/severe) binary classification in private wider data sets comprising 80,000 to 120,000 fundus images.

There are many traditional strategies for DED diagnosis and classification [8, 19, 51]. Most techniques use neural networks, mathematical morphology, region of interest techniques, pattern recognition, clustering of fuzzy C-means, and Gabor filter techniques. For example, Chaudhuri et al. [8] uses 2D matched filters, to detect the blood vessels present in the retina. Vallabha et al. [51] uses Gabor filter bank outputs to identify the mild, moderate, and extreme stages of retinopathy, the automated detection and classification of abnormalities present in the vascular network are carried out.

There are numerous methods suggested for optic disc detection. Principal component analysis (PCA) is one of the methods by which clustering of brighter pixels derives the candidate regions for the optical disc. Noronha et al. [32] used Hough Transform for Optic Disc detection. In the detection of exudates, a neural network-based approach is used by Gardner et al. [14], For the detection of exudates, a fuzzy C-means clustering method employed by bezdek et al. [5] and a computational intelligence-based approach are used by Osareh et al. [33]. The automatic classification of normal, mild, moderate, severe, and proliferative diabetic retinopathy is carried out by measuring the areas of several characteristics, such as haemorrhages, microaneurysms, exudates, and blood vessels classified by the support vector machine [2].

However, the accuracy metrics for the diagnosis of four categories of DR, i.e., no DR, mild, moderate, and severe, are significantly based on disease-grade selection ratios. Although the no DR and severe stages are likely to achieve high sensitivity, the mild and moderate recall levels are often deficient. Research studies using publicly available datasets reveal difficulties in detecting early-stage DEDs.

## Methods

The research’s overarching objective is to improve the performance of early detection of DED from fundus images through the empirical assessment of image preprocessing and classification improvement techniques. The related objectives can be described as follows:

- Implementing traditional image processing techniques such as (i) Image Enhancement, (ii) Image Augmentation, (iii) Image Segmentation;
- Implementing various hyperparameters and evaluate their effect on CNN models’ performance;
- Evaluate the accuracy obtained by pre-trained CNN models: *ResNet50*, *VGG-16*, and *Xception* with original and preprocessed fundus images;
- Developing a new CNN model to train preprocessed fundus images for classification accuracy improvement;
- Evaluate the results of pretrained and new CNN model by performance metrics.

**Fig. 2:**
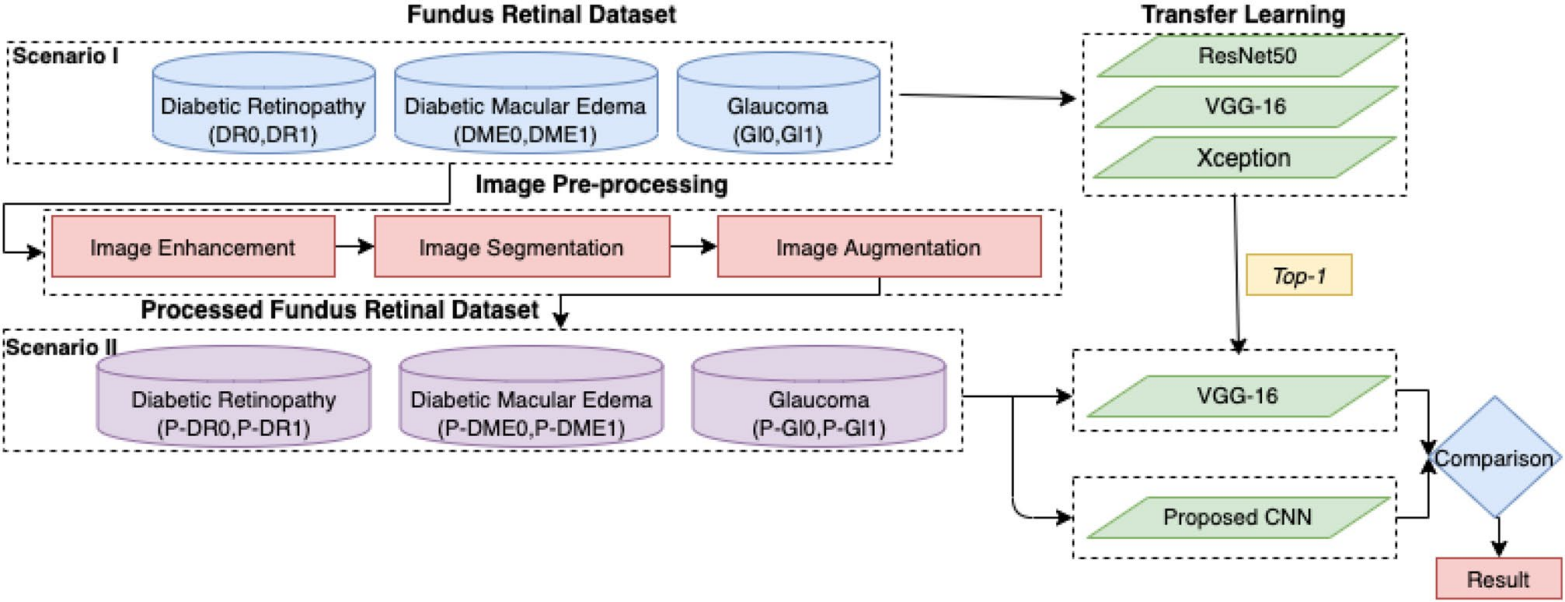
The high-level process pipeline

To high-level process, a pipeline is shown in Fig. 2 which demonstrates the importance of this workflow. In which, we experimented with the raw retinal fundus dataset employing three pre-trained models such as, (ResNet50, VGG-16, Xception) and obtained the highest performing model. We used traditional image processing algorithms to our raw fundus images and then trained this dataset with best performing model from the previous experiment. We also trained preprocessed images with CNN architecture from scratch. Finally, we compared the results to check preprocessed images improved the performance accuracy of the models or not.

### Data Collection

Data was collected from publicly accessible sources, i.e., Messidor, Messidor-2, DRISHTI-GS, and Retinal Dataset from GitHub. This section explains the data sets used in these articles. The labeling of each image is generated by the ophthalmologist. Depending on the number of hemorrhages, microaneurysms, and the presence of neovascularisation, each image is classified as one of three lesion grades. *Messidor Dataset* has been formed to promote computer-assisted DED studies. Messidor database collected 1200 retinal fundus images of the posterior pole from three departments of ophthalmology using a 3CCD color video camera placed on a Topcon TRC NW6 non- retinograph with a $45^{\circ }$ field of view (FOV). The medical experts offered two diagnostics for each image: *Retinopathy grade* and * Macular edema risk*. *Messidor-2 Dataset* is a publicly accessible dataset used by other individuals to evaluate DED algorithms’ performance. Messidor-2 comprises 1,748 colour retina images of 874 subjects. Messidor-2 varies from the actual Messidor dataset of 1200 images and ensures that it has two images for each subject, one for each eye. Using the previously published ICDR and DME gradings, Messidor-2 provided four disease rates for each subject. *DRISHTI-GS Dataset* [44] There are 101 retinal images in the Drishti-GS1 dataset with 31 normal images and 70 glaucoma lesion images. Due to the limited images obtained from DRISHTI-GS, we considered the glaucoma dataset from GitHub 1*Retina Dataset* which contained 100 retinal images indicating glaucoma lesions. Therefore, improvement in the imbalance dataset can cause the improvement of the predictive model [52]. Thus we performed undersampling of the dataset in this study and selected 100 images from each class to perform our experiment.

### Image Pre-processing

The preprocessing step is used to eliminate noise/variation in the retinal fundus image and improve the quality and contrast of the image. Apart from contrast enhancement and noise reduction, the preprocessing step can be used for image normalization and non-uniform intensity correction to eliminate artifacts and increase the accuracy of the process steps. Furthermore, DED features are localized, extracted, and segmented from fundus images for further classification in pre-trained models. The preprocessing techniques utilized in this article are briefly discussed in this section.

**Fig. 3:**
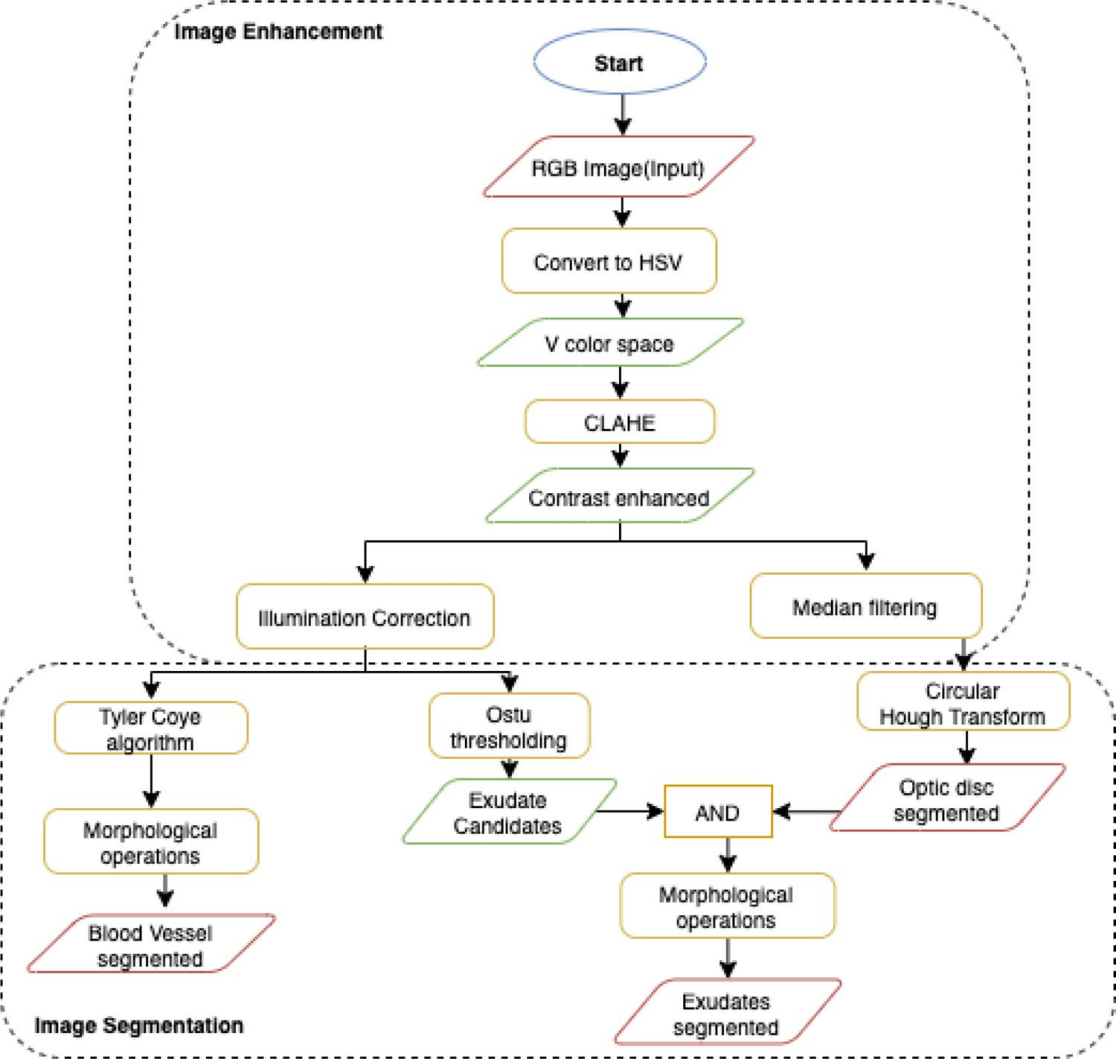
The flow chart

#### Image Enhancement

To enhance the original images’ appearance and information value before processing, we used image enhancing techniques, popular techniques such as contrast enhancement, illumination correction. *Contrast enhancement*: Contrast limited adaptive histogram equalization (CLAHE) [56] is utilize to improve the visibility of images. CLAHE is an adapted part of the Adaptive Histogram Equalization (AHE) process. In this method, the enhancing function is introduced to all neighborhood pixels, and the transformation function is derived. This is distinct from AHE for its limited contrast. In CLAHE, the contrast of the image is improved by implementing Contrast limited histogram equalization (CLHE) to small data areas called tiles rather than the entire image. The resulting adjacent tiles are then perfectly stitched back utilising bilinear interpolation. CLAHE applied to greyscale retinal images. The ’cliplimit’ function is applied to limit noise in an image. Create gray level mapping and clip the histogram. In the contextual area, pixel numbers are divided equally at each grey level so that the average number of pixels is grey as follows:

1 $$\begin{aligned} n_{avg}={n_{CR-x_{p}}*n_{CR-y_{p}}\over n_{gray}} \end{aligned}$$

Where, $n_{avg}$= average number of pixels, $n_gray$= number of gray level in contextual region

${n_{CR-x_{p}}}$= number of pixels in *x* direction of contextual region

${n_{CR-y_{p}}}$= number of pixels in *y* direction of contextual region After that calculate the actual cliplimit.

2 $$\begin{aligned} n_{CL} =n_{CLIP}*n_{avg} \end{aligned}$$

CLAHE [56] is an useful technique in biomedical image processing because it is very effective at making the normally important salient sections more accessible.

*Illumination correction* This preprocessing method aims to reduce the scenario effect caused by uneven illumination of retinal images [13]. Every pixel intensity is calculated using the following equation:

3 $$\begin{aligned} p^{\prime } = p + \mu _{D} - \mu _{L} \end{aligned}$$

Where* p*,* p*’is the initial and the latest pixel size values, respectively, $\mu$D is the desired average intensity, and $\mu$L is the local average intensity [54]. Microaneurysms forming at the surface of the retina are enhanced by this method.

#### Image Segmentation

To build an effective deep learning-based classification system for detecting mild DED, we need to consider the importance of the architecture of the network as well as the importance of input data. To obtain an efficient results, input images plays significant role. In retinal fundus images, variability such as the number of images, luminosity, contrast, and anatomical features determines the forthcoming result of the automatic disease detection algorithm. Therefore, features segmentation enhances the value of the images for classification and contribute for better accuracy. The process and the necessary theory is explained in the following sections.

*Blood Vessels Extraction* For detecting early stages of DR, the blood vessels are one of the most significant anatomical feature in retinal images. Thus retinal blood vessels segmentation is performed with following steps: (1) image enhancement; (2) Tyler Coye algorithm [10] and (3) morphological operation for further improvement in results.

We performed image enhancement techniques as mention above, green channel of the RGB color space presents better contrast between vessels network and background. The variation of contrast and luminosity in a background of a fundus image, can be estimate using method introduced by Zuiderveld [56] and Youssif et al. [54]. After, contrast and luminosity adjustment, ISODATA used in Tyler Coye algorithm is used for extracting threshold level. After tyler coye algorithm, morphological operation (erosion and dilation) used for further enhancement. Using these two essential fundamental operations we reduce noise or remove of gaps in the background and foreground. Erosion is a procedure used to eliminate or spike the edge of the area, which is represented in the following equation.

4 $$\begin{aligned} A \ominus B=\{p\vert B_{p}\subseteq A\} \end{aligned}$$

Dilation is a procedure employed to broaden the rim of the background or foreground image configuration. This procedure is widely used to fill a gap, that can be defined in the following equation.

5 $$\begin{aligned} A\oplus B=\{x\vert B_{x}\cap X\ne 0\} \end{aligned}$$

Closing is to perform the dilation, followed by erosion, to create a relation between each pixel of the image in order to bring it closer to one another. This procedure can be defined in the following equation,

6 $$\begin{aligned} A\cdot B=(A\oplus B)\ominus B \end{aligned}$$

Where, $\oplus$ denote the dilation; $\ominus$ denote the erosion; *A* = Structuring element and *B* = the erosion of the dilation of that set. However, several gaps remain in Tyler Coye algorithm. This morphological process is to fill these small gaps in order to cover some of the required regions of the blood vessels.

*Optic Disc Detection and Extraction*: Glaucoma occurs when optic nerve is damaged. So, the segmentation of the optic disc (OD) helps to view the clearer anatomical changes in optic nerve. Figure 1 shows an fundus image of an eye from our collected data set with anatomical parts showing OD. To segment the OD we applied following steps: (i)image enhancement, (ii) Circular Hough Transform (CHT) to detect circular object, (iii) median filter to reduce noise, and (iv) optic disc segmentation using the threshold values. Image processing attempts to improve and increase the quality of the retinal fundus image in order to enable the identification of clinical features for DED. Flowchart of the image processing and image segmentation approach is depicted in Fig. 3. CLAHE can not be employed in the entire image, but only on a specific area ’tile’ of the image.

Image enhancement calculation is adjusted on the basis of the user-specific maximum contrast-rate level by setting its rate to *l*,0 $\le$ *l* $\le$ *l* [46]. Further contrast enhancement is performed in those images which has low contrast estimated by

7 $$\begin{aligned} \phi (i, j)=\left( \frac{\mu (i, j)-\varDelta }{\delta -\varDelta }\right) (\varGamma -1) \end{aligned}$$

Where, $\phi (i,j)$ and $\mu (i,j)$ are pixels after transformed and pixels before transformed in (*i*, *j*) coordinate, respectively; $\varDelta$ is maximum pixel value; $\delta$ is minimum pixel value of input image and $\varGamma$ is maximum value of gray scale.

The median filtering has a strong noise reduction efficiency and it is very common in image processing for noise removal. Mean filtering replaces the pixel value in the middle of the sliding window with the median value of the pixels in the window. Mathematically median filtering is represented in,

8 $$\begin{aligned} f(x, y)=median_{(s, t)\in S_{xy}}\{g(s, t)\} \end{aligned}$$

Segmentation is a pixel classification method for extracting objects or segmenting regions with a similar attributes from the background [41]. Therefore, we used the Circular Hough Transform (i.e. CHT) method for optical disc detection. The CHT method is often used to identified circular shape in an image. The key benefit of the CHT approach is that it is sensitive of differences in feature specification descriptions as well as largely unaffected by image noise. The CHT is provided by given equation:

9 $$\begin{aligned} (x-a)^{2} + (y-b)^{2} = c^{2} \end{aligned}$$

The procedure to detect circles involves the following steps:(i) we obtain a binary edge map of the image; (ii) values for *a* and *b* are set; (iii) obtain the value of *c* radius that satisfies Eq. 7; (iii) adjust the accumulator corresponding to (a,b,c); (iv) change the values for *a* and *b* within the scope of interest and return to Phase (iii).

*Exudate localization and Detection*: Exudate in two-dimensional retinal images acquired via a digital fundus camera, usually appear as bright area with varying scale, brightness, position and form. Precise exudate segmentation is a difficult activity given the large variety of scale, intensity, contrast and shape. It comprises of three major processing stages: (1) image enhancement; (2) optic disc detection and removal; (3) blood vessel removal; and (4) exudate extraction. When exudate are acquired from the mild dataset, the classification of the DME can be performed according to the grading criteria mention in the messidor dataset. Early DME can be diagnose early by detecting the presence of exudates in fundus images. Figure 1 shows the exudates formation in the macular region. After *optic disc detection and removal* performed, Otsu thresholding is applied to obtain candidate areas of exudates. Threshold value *T* relying on the input image is estimated by Ostu method, automatically. First, intensity value *i* of histogram is calculated using Eq. (11),

10 $$\begin{aligned} p(i)=\frac{n_{i}}{N}, p(i)\ge 0,\sum _{1}^{256}p(i)=1 \end{aligned}$$

The number of pixel images *N* and the number of pixels $n_i$ with *I* intensity. Subject weight and background are described in (12) and (13).

11 $$\begin{aligned} w_{1}(t)= &  \sum _{i=1}^{t}p(i) \end{aligned}$$

12 $$\begin{aligned} w_{2}(t)= &  \sum _{i=t+1}^{L}p(i)=1-w_{1}(t) \end{aligned}$$

Here, the number of the gray level is *L*. The mean of the object and the background is then determined using Eqs. (14) and (15)

13 $$\begin{aligned}&m_{1}(\mathrm {t})=\sum _{i=1}^{t}i.p(i)/w_{1}(t) \end{aligned}$$

14 $$\begin{aligned}&m_{2}(t)=\sum _{i=1}^{t}i.p(i)/w_{2}(t) \end{aligned}$$

Hence, Variance is estimated by Eqs. (16, 17), while total of variance is expressed in Eq. (18) as follows.

15 $$\begin{aligned} \sigma _{1}^{2}(t)= &  \sum _{i=1}^{t}(1-m_{1})^{2}.\frac{p(i)}{w_{1}(t)} \end{aligned}$$

16 $$\begin{aligned} \sigma _{2}^{2}(t)= &  \sum _{i=t+1}^{t}(1-m_{2})^{2}.\frac{p(i)}{w_{2}(t)} \end{aligned}$$

17 $$\begin{aligned} \sigma ^{2}(t)= &  \sigma _{w}^{2}(t)+\sigma _{B}^{2}(t) \end{aligned}$$

Here, $\sigma 2w$ is called as within-class variance (WVC) that is expressed in Eq. (19), while $\sigma 2B$ called between-class variance (BVC) that is expressed in Eq. (20). WVC is the amount of individually class variance that has been weighted with probability of each class. Average total is calculated using Eq. (21). Threshold value can be obtained from minimisation of WVC or maximisation of BVC, but BVC has less computation time.

18 $$\begin{aligned} sigma_{w}^{2}(t)= &  w_{1}(t).\sigma _{1}(t)^{2}+w_{2}(t).\sigma _{2}(t)^{2} \end{aligned}$$

19 $$\begin{aligned} \sigma _{B}^{2}(t)= &  w_{1}.\ [m_{1}(t)-m_{T}]^{2}+w_{2}.\ [m_{2}(t)-m_{T}]^{2} \end{aligned}$$

20 $$\begin{aligned} m_{T}= &  \sum _{i=1}^{N}i.p(i) \end{aligned}$$

Morphological is a set of discrete coordinates that related to pixel object of image that involves logical operation, such as “or” and “and”. Opening operation aims to refine object contour and repair object contour with eliminated pixel area that smaller than structure element. Opening operation is expressed in Eq. (22).

21 $$\begin{aligned} AoB=({A}\Theta B)\oplus B \end{aligned}$$

### Image Augmentation

Deep learning models perform well with high-volume training data [26, 35]. Therefore, data augmentation comprises a collection of techniques that improve the quantity of training data without actively acquiring new data. Thus, image augmentation algorithms addressed in this paper include geometric transformations such as flipping, rotation, mirror, and cropping. We used Keras *ImageDataGenerator* class for real-time image augmentation, which ensures that the selected model will obtain variations of the images at every epoch. The advantage of using *ImageDataGenerator* class in our work is transformed images will not add to the range of original images, which avoid overfitting the selected model.

### Transfer Learning

In this research, we are using CNNs-based transfer learning to implement the DED retinal fundus image classification. To accomplish the absolute best classification outcomes, we explore pre-trained CNN model transfer learning techniques. The precise details of the pre-trained models will be presented in this section.

According to Pan et al. [34] transfer learning is defined as; *D* = ${ \varPhi , P(X) }$ with $X={x_1,x_2,...,x_n}$ $\epsilon$ $\varPhi ,$ where, *D* is domain, $\varPhi$ is refer to a feature space and *P*(*X*) marginal probability distribution. Given, *T* = ${Y,F(*)}$ where, *T* is given task, *Y* is refer to a label space and $F(*)$ is an objective predictive function that is learned from the feature vector and label pairs. Specifically, given a source domain $D_s$ with learning task $T_s$ and a target domain $D_t$ with learning task $T_t$, then transfer learning is the process of improving the learning of the target predictive function $F_t( *)$ in $D_t$ based on the knowledge learned from source domain $D_s$ and learning task $T_s$, where $D_t$ $\ne$ $D_t$, or $T_s$ $\ne$ $T_t$. It should be noted that the single source domain described above can be expanded across multiple source domains.

The concept behind the transfer learning for the classification of images is that if a network is typically trained on a broad scale and enough data set (e.g., ImageNet), it can effectively train in the particular target task, which has fewer labeled examples than the pre-training dataset. One can benefit from these learned feature maps without training a large model from scratch on a large dataset.

In this paper, we will customize pre-trained models in two ways: *(1) Feature Extraction*, features learned from the source task to extract useful features from the target task. We added a new classifier, which can be trained from scratch to the top of the pre-trained network to modify the features maps initially learned for the sample. *(2) Fine-Tuning*, unfreeze some of the last layers of the frozen base network and collectively train the last layers of the base network and the newly added classifier layers. This helps to “fine-tune” the higher-order character representations in the base network to make them more appropriate to the target task. We fine-tune three pre-trained CNN networks(Xception, VGG-16, and DenseNet21) to implement DED image classification. Three CNN pre-trained network on ImageNet and their characteristics are described in Table 1.

**Table 1:** Three CNN models pre-trained on ImageNet and their characteristics.* Source*: https://keras.io/applications)

Model	Size	Top-1 accuracy*	Top-5 accuracy*	Parameters	Depth**	Reference
Xception	88 MB	0.790	0.945	22,910,480	126	Chollet [9]
VGG16	528 MB	0.713	0.901	138,357,544	23	Simonyan and Zisserman [43]
DenseNet21	33 MB	0.750	0.923	8,062,504	–	Huang et al. [20]

*The top-1 and top-5 accuracy refers to the model’s performance on the ImageNet validation dataset.

**Depth refers to the topological depth of the network. This includes activation layers, batch normalisation layers etc

### Proposed CNN Model

**Fig. 4:**
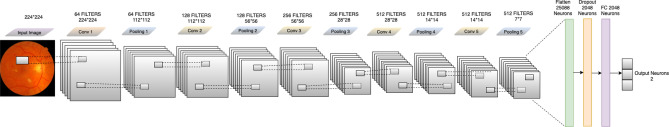
The proposed CNN model

CNN’s are the most popular deep learning algorithms which train the medical images for the classification of medical image abnormalities [29]. The explanation for this is while analyzing input images, CNN preserves distinctive features. Spatial relationships, such as where the blood vessels start rupturing or how yellow fluid starts accumulating near the macular region, are of primary importance in retinal images, as we discussed above. The framework of the process is shown in Fig. 4 and Table 2 shows the selected hyperparameters. There are five convolution layers in this proposed CNN model, which take as its input a retinal fundus image tensor of $244\times 244$.

**Table 2:** Hyper-parameters of the build CNN model and preferred weights in this study

R1	R2	R3	R4	R5	R6	R7	R8
CNN	224*224	RMSprop	32	10-fold	3e-4	BCE	50

R1—model, R2—image size, R3—optimizers, R4—mini batch size, R5—cross validation, R6—initial learning rate, R7—loss function, R8—Epoch, *BCE*—binary cross-entropy

Subsequently, the first convolution layer uses $5\times 5 \times 3$ kernel filters with stride $1\times 1$, and a total of 64 such filters are employed. The next layer, which receives the output from the first layer, is a max-pooling layer with $2\times 2$ stride, reducing the input to half of its size $112\times 112$. For all layers, the output from the pooling layer passes through the ReLU activation feature. The nonlinear output obtained is now fed into the next convolution layer with $5\times 5 \times 64$ with 128 filters, and the stride value is the same $1\times 1$. The obtained output pass through a max-pooling layer with the same $2\times 2$ strides, which again reduced the input to half of its size $56\times 56$. After the output pass through ReLU activation, it is fed into the third convolution layer with 256 filters and the kernel size $5 \times 5 \times 128$ with $1\times 1$ stride. The output is passed to a max-pooling layer, which results in a tensor of shape $28 \times 28$. Again the output pass through ReLU activation, fed into the fourth convolution layer with 512 filters and kernel size $5\times 5 \times 256$ and with the same stride $1\times 1$. The output from the fourth convolution is max-pooled to a size of $14 \times 14$. After ReLU activated and it is pass to a fifth convolution layer with 512 filters and $14\times 14\times 512$ kernel size to accommodate the output of all the filters from previously configured layers, and max-pooling of output from that layer with a stride of size $2 \times 2$ produces an output of size $14 \times 14$. Now the resulting tensor has the shape $7 \times 7 \times 512$. The obtained tensor is flattened with 25,088 neurons. The weighed values that emerge as neurons demonstrate the proximity to the symptoms of COVID-19. The dropout layer is applied here to drop values to handle network overfitting. In our work, we used a dropout rate of 0.5 during training. The fully connected layer converts the tensor with 25,088 neurons to 64 neurons and adds ReLU activation to the output. A tensor with 64 neurons is the product of the fully connected layers; these 64 neurons are translated into neuron counts equal to the number of categories to which the retinal image belongs, healthy, diabetic retinopathy, glaucoma, and diabetic macular edema.

### Evaluation Criteria

**Fig. 5:**
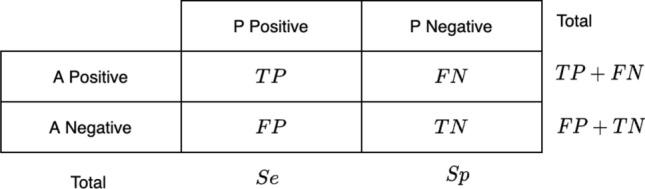
Confusion matrix: a positive = actual positive, a negative = actual negative, P positive = predicted positive, P negative = predicted negative, *TP* true positive, *FN* false negative, *FP* false positive, *TN* true negative, Se = TP + FP, Sp = FN + TN

Different metrics have been used to evaluate the efficiency of the highest performing DL model. To calculate the true or false classification of the DED diagnosed in the fundus images evaluated as follows. Initially, the cross-validation estimator [45] is adopted and plotted in a confusion matrix as shown in Fig. 5. The confusion matrix has the following four predicted outcomes. True Positive (TP) has been identified with the right diagnosis and a variety of abnormalities. True Negative (TN) is an erroneously calculated number of periodic instances. False positives (FP) are a set of periodic instances. The following performance metrics are used to calculate the values of possible outcomes in the confusion matrix.

*Accuracy* Accuracy is an essential metric for the evaluation of the results of DL classifiers. It is a summary of the true positive and true negatives divided by the confusion of the matrix components’ total values. The most accurate model is an excellent one, but it is imperative to ensure that symmetric sets of data with almost equal false positive values and false negative values. Thus, the elements of the confusion matrix mentioned above will be calculated to evaluate the effectiveness of our proposed classification model for the DED dataset.

22 $$\begin{aligned} \rm{Accuracy}\;(\%) = \frac{\rm{TP} + \rm{TN}}{\rm{TP}+\rm{FN}+\rm{TN}+\rm{FP}} 100\%. \end{aligned}$$

*Sensitivity (Recall):* sensitivity is measured as the number of accurate positive predictions divided by the sum of positive. The best sensitivity is 1.0, whereas the worst is 0.0. We calculate sensitivity using following equation;

23 $$\begin{aligned} \rm{Sensitivity} = \frac{\rm{TP}}{\rm{TP}+\rm{FN}} \end{aligned}$$

*Specificity:* specificity is measured as the number of correct negative predictions divided by the sum of negatives. The best specificity is 1.0, whereas the worst is 0.0. We calculate sensitivity using the following equation;

24 $$\begin{aligned} \rm{Specificity} = \frac{\rm{TN}}{\rm{TN}+\rm{FP}} \end{aligned}$$

## Experiments and Results

All the experiments are implemented using MatLab, Python, Keras library1, with TensorFlow2 as a back-end and Python 3.8 programming language in jupyter notebook with a processor 2.3 GHz Intel Core i9 and RAM of 16 GB 2400 MHz DDR4 with Intel UHD Graphics 630 1536 MB. The training/testing data split was set at 80/20. The segregated generic selection was conducted to ensure an approximately equal distribution of the class. Mini-batch size was set to 32, and the cross-entropy loss function was chosen due to its suitability for binary classification tasks. The Optimiser was set as default (Adam) and RMSprop for build CNN. The standard performance evaluation metric accuracy, sensitivity, and specificity of the test dataset were used to validate results.

We compared and analysed performance accuracy among three distinct pre-trained deep learning models with the new build CNN model in this study. The three pre-trained models, namely; *Xception*, *VGG16*, and *DenseNet21* and five-layered convolutional model have been evaluated in terms of test data set accuracy (Table 1). The pre-trained models adopted for this research were trained and tested by large-scale ImageNet data, covering a wide range of categories such as cars, animals, flowers, etc. Models acquire excellent performance image classification for objects while demonstrating a limitation in their application to narrow product areas, such as medical lesion (DED) detection. The prognosis of pathological indications in the retinal fundus images depends upon various complex characteristics and lesion localization in the retinal fundus image. There is a new representation of the input image in each CNN layer by progressive extraction of the most distinctive features. For instance, the first layer is capable of learning edges, while the last layer can identify a lesion as a DED classification feature. As a result, the following scenarios were considered in the experiments: Region of Interest such as blood vessels, macular regions, and the optic disc has been detected, localized, and segmented.

We have employed a combination of multiple traditional image segmentation algorithms for each phase in the proposed system. All of these algorithms provided effective results in the segmentation of the region of interest. We performed a series of procedures to build a high-performance system, such as image enhancement, blood vessel segmentation, identification and then extraction of optic discs, extraction of macular region, blood vessels removal, elimination of optic discs, extraction of features, and classification of features. After segmentation, the size of the images has been optimized to a suitable size following the input specifications of each network. To minimise the risk of model overfitting, the imbalance dataset was augmented using real-time augmentation *ImageDataGenerator* class from Keras. The fine-tuning was used for pre-trained models after eliminating and re-training *n* layers (*n* was CNN layer-dependent). The final output acquired for each model was used for comparison in terms of percentage accuracy are represented in Tables 3 and 4. VGG16 classification surpassed the other two fully-trained deep learning models Xception and DenseNet121. Similarly, among all pre-trained models, new build CNN model using preprocessed retinal images performed well. Tables 5 and 6 compare the accuracy of results. Build CNN accuracy surpasses all the model used for classifications.

**Fig. 6:**
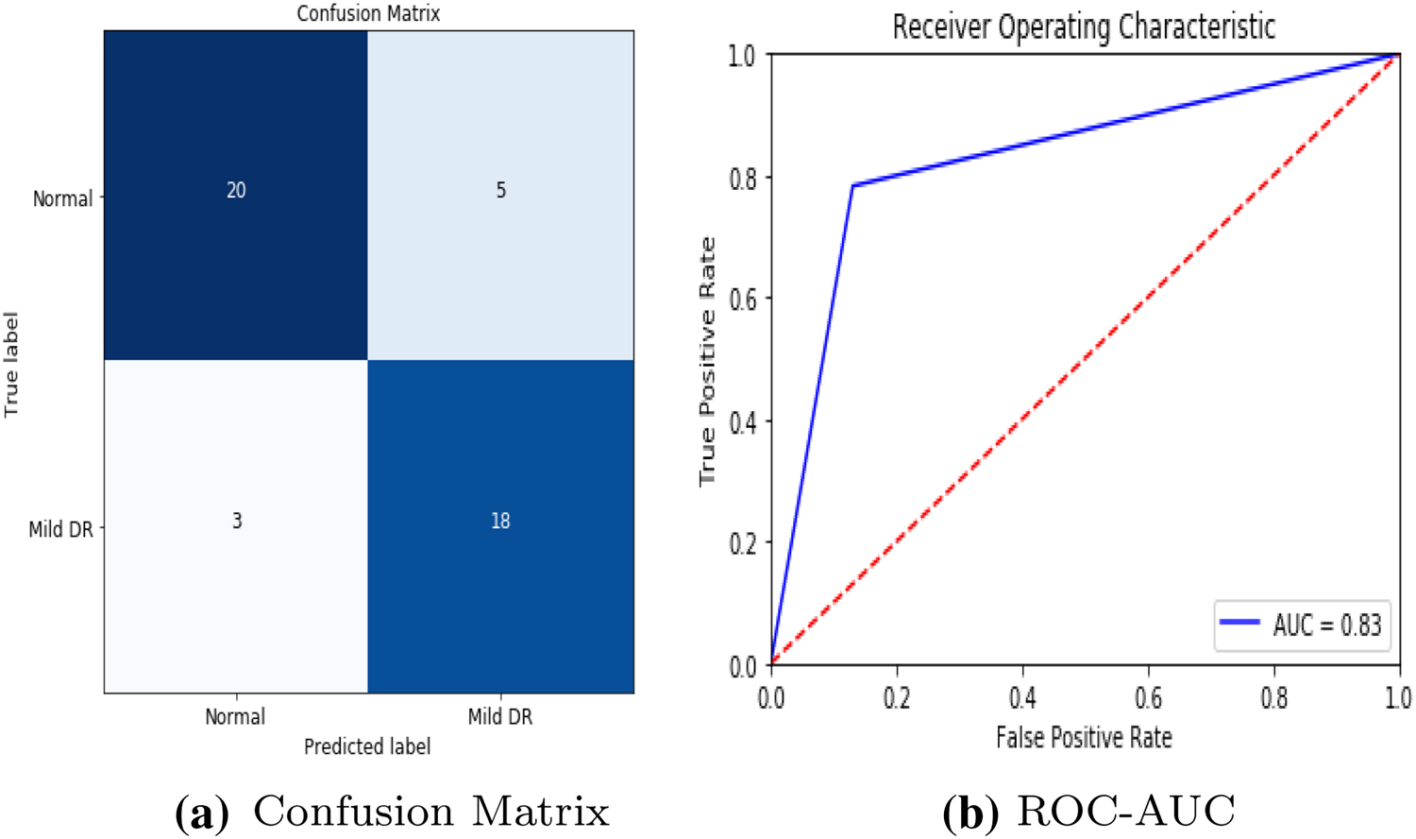
VGG16 model performance in diabetic retinopathy

**Fig. 7:**
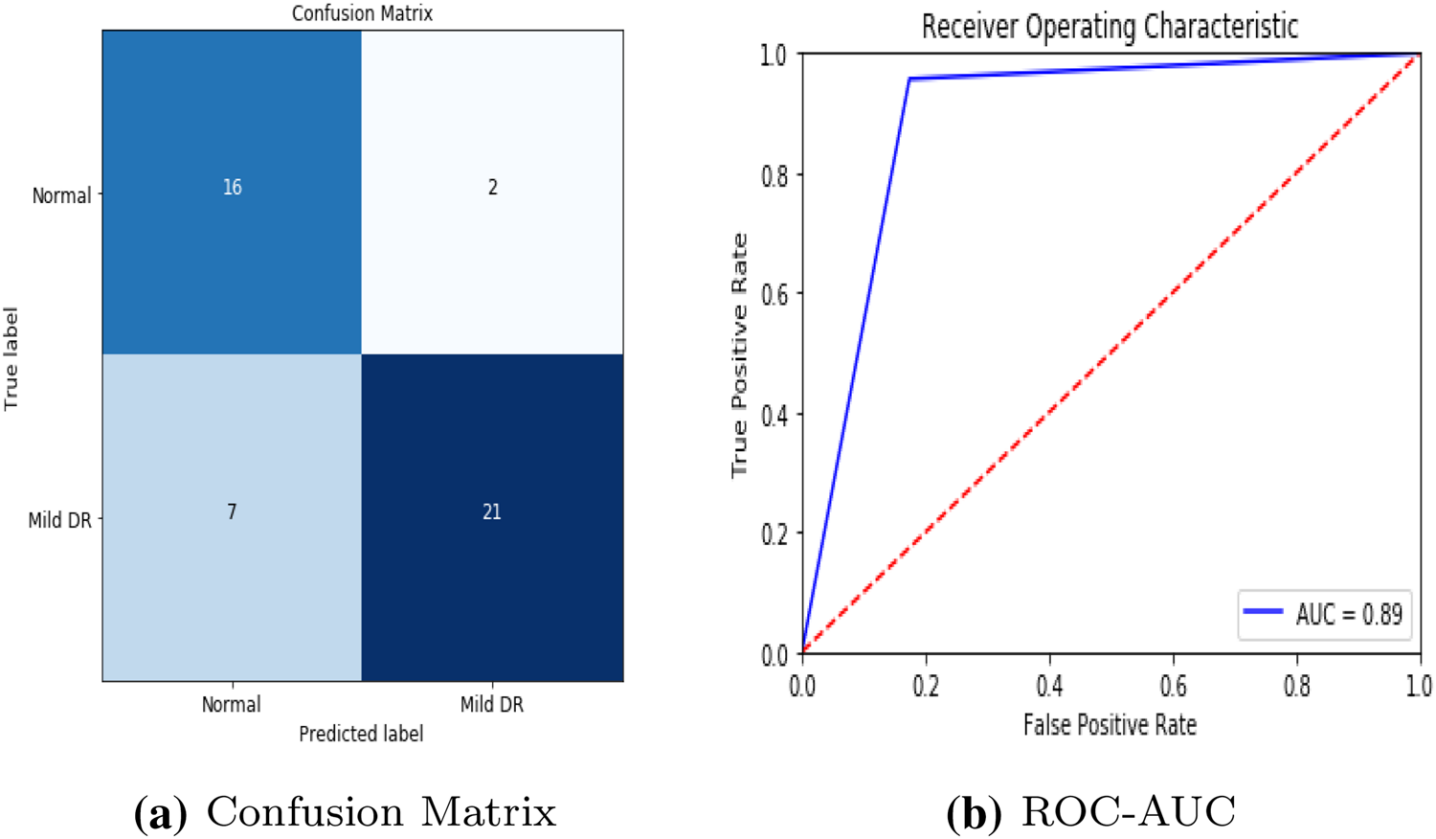
VGG16 model performance in diabetic macular edema

**Fig. 8:**
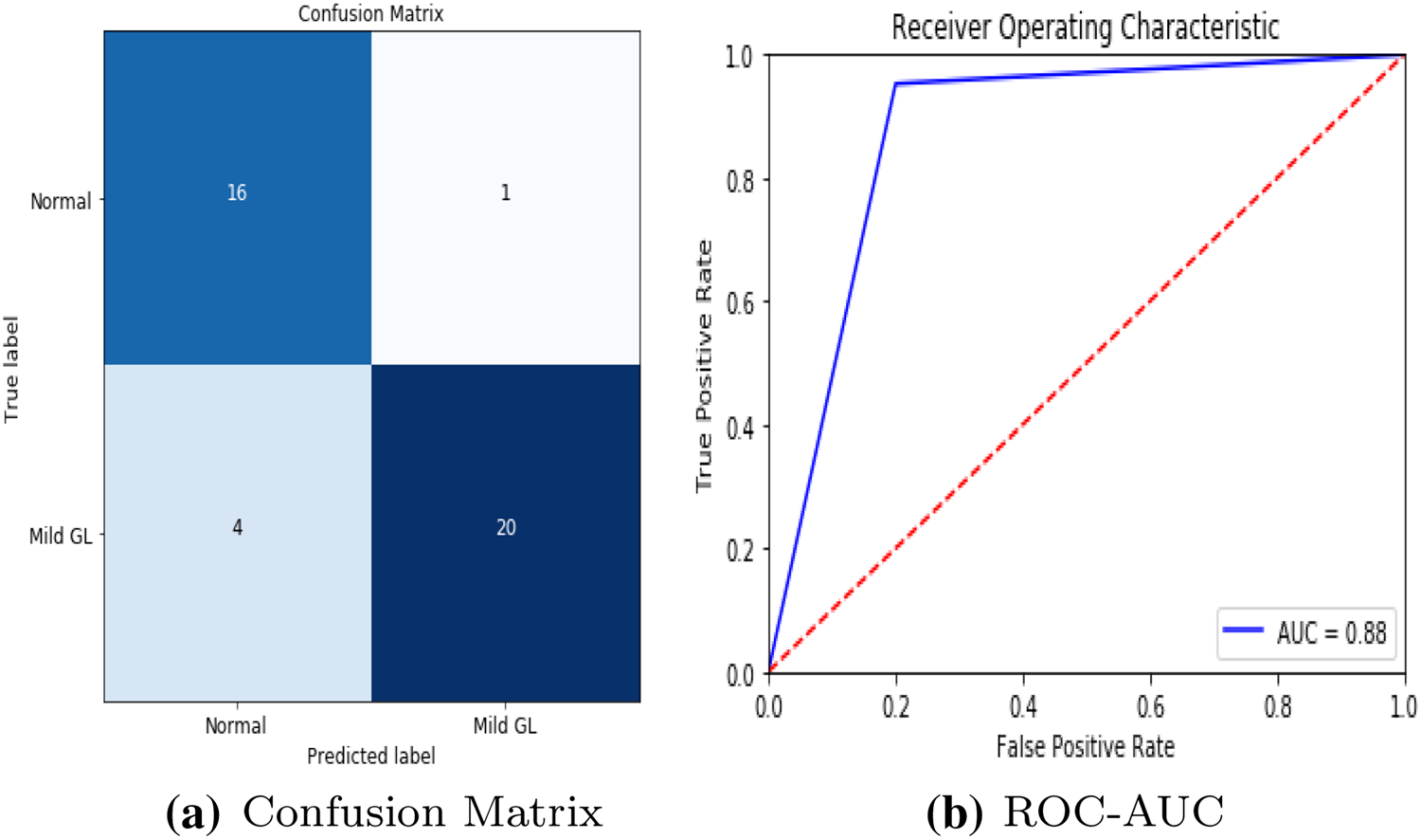
VGG16 model performance in Glaucoma

**Fig. 9:**
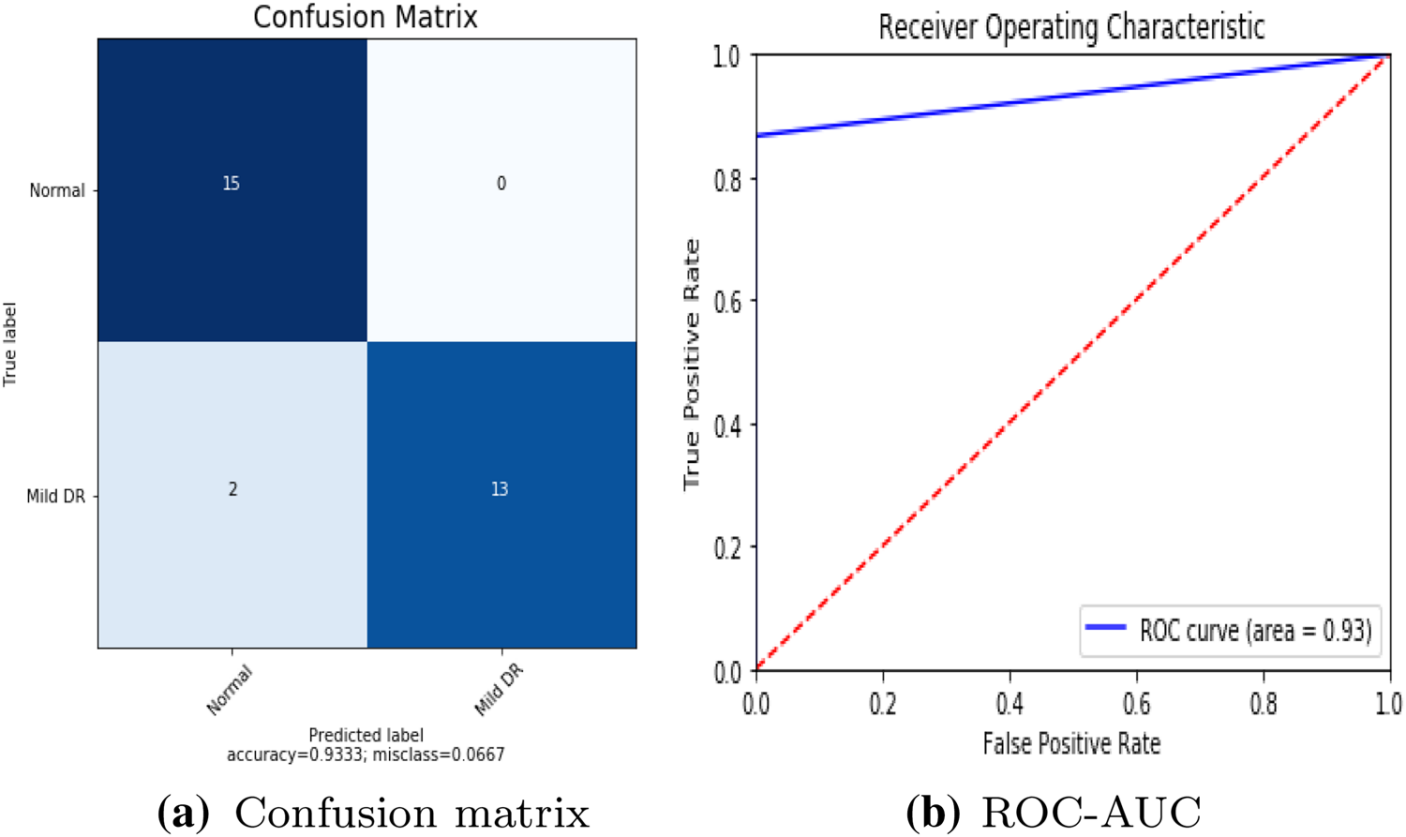
Build CNN model performance in diabetic retinopathy

**Fig. 10:**
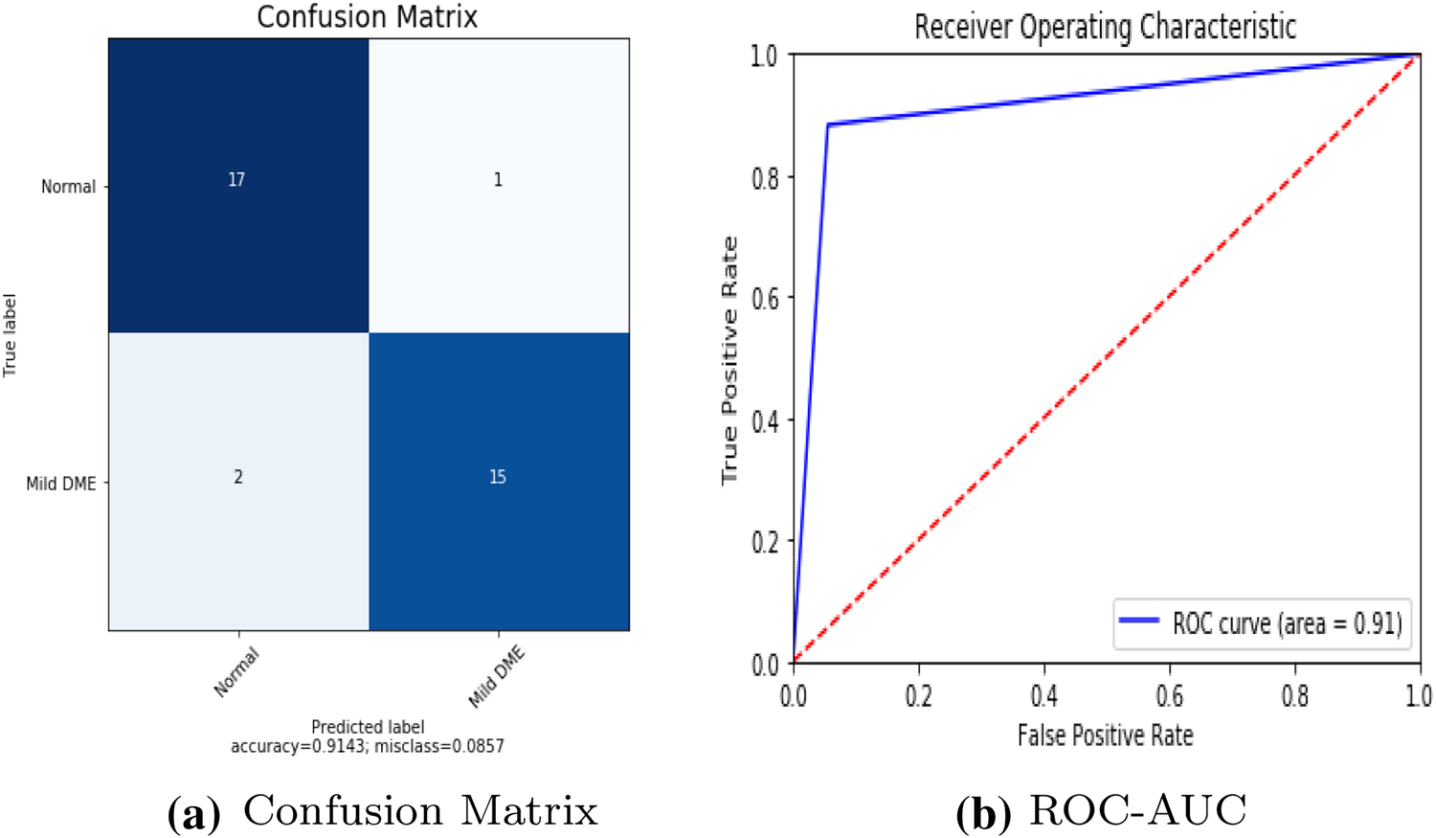
Build CNN model performance in diabetic macular edema

**Fig. 11:**
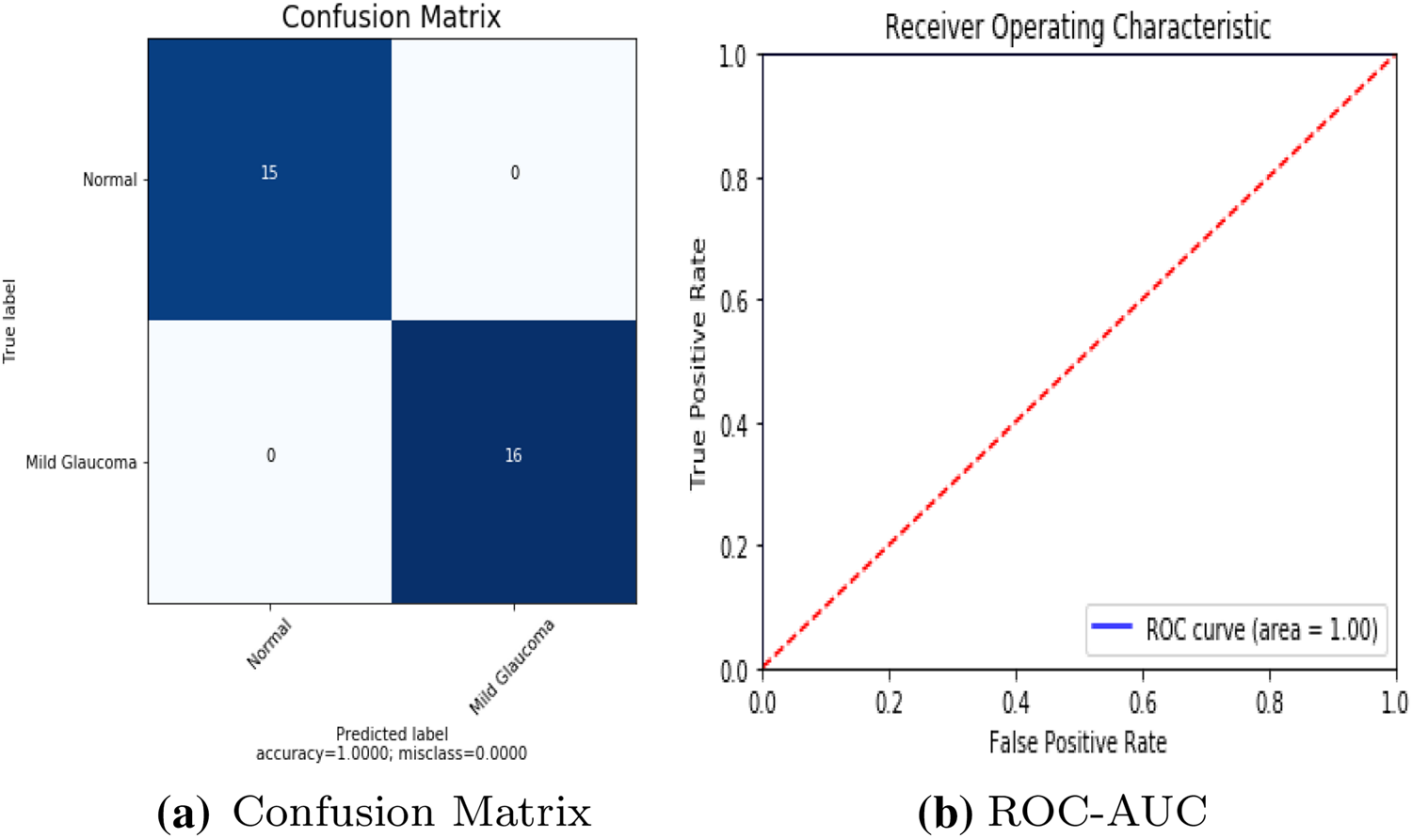
Build CNN model performance in Glaucoma

To detect retinal anomalies, we developed more general screening classification models. The confusion matrix and ROC curves of each pre-trained deep learning model and a build CNN model for binary classification of healthy and other DED disease status are shown in Figs. 6, 7, 8, 9, 10 and 11.

**Table 3:** Average performance of the models on original images

DED	Model	Accuracy (%)	Sensitivity (%)	Specificity (%)	Precision (%)
Normal /mild DR	Xception	60.87	67	58	43
	VGG16	**80.43**	**76.92**	**85**	**74**
	DenseNet121	56.67	71.43	53.85	96
Normal /mild DME	Xception	62.07	65	60	52
	VGG16	**85.79**	**90**	**81**	**78**
	DenseNet121	51.72	100	51	28
Normal /mild GL	Xception	63.41	85.71	58.82	95
	VGG16	**87.80**	**94.12**	**83.33**	**95**
	DenseNet121	80.49	77	84	76

Bold values indicate VGG16 classification surpassed the other two fully-trained deep learning models

*DED* diabetic eye disease, *DR* diabetic retinopathy, *DME* diabetic macular edema, *Gl* Glaucoma

**Table 4:** Average performance of the VGG16 model on pre-processed images

DED	Model	Accuracy (%)	Sensitivity (%)	Specificity (%)	Precision (%)
Normal /mild DR	VGG16	83.43	86	85.71	78
Normal /mild DME	VGG16	89.13	85	95	96
Normal /mild GL	VGG16	88	95	90	90

*DED* diabetic eye disease, *DR* diabetic retinopathy, *DME* diabetic macular edema, *Gl* Glaucoma

**Table 5:** Average performance of the new proposed model on original images

DED	Model	Accuracy (%)	Sensitivity (%)	Specificity (%)	Precision (%)
Normal /mild DR	CNN	63.33	53.33	73.33	61
Normal /mild DME	CNN	82.86	83.33	82.35	82
Normal /mild GL	CNN	96.77	100	93.75	100

*DED* diabetic eye disease, *DR* diabetic retinopathy, *DME* diabetic macular edema, *Gl* Glaucoma

**Table 6:** Average performance of the new proposed model on pre-processed Images

DED	Model	Accuracy (%)	Sensitivity (%)	Specificity (%)	Precision (%)
Normal /mild DR	CNN	93.33	100	86.67	100
Normal /mild DME	CNN	91.43	94.44	88.24	94
Normal /mild GL	CNN	100	100	100	100

*DED* diabetic eye disease, *DR* diabetic retinopathy, *DME* diabetic macular edema, *Gl* Glaucoma

## Discussions

This research is a study of binary classification deep learning algorithms to identify three mild diabetic eye diseases automatically. This research has shown that the complexity of the deep learning algorithms arises from the quality and quantity of data (fundus images), not from the algorithm. In this research, we used publicly available annotated data (fundus images). For a computer-aided clinical application, more robust, practical, and realistic results can be obtained using labeled hospital fundus images. Indeed, this paper recommends that the automatic classifier strive to classify against the binary classification of at least average, DR, DME, and GL due to each disease’s significance. These three diseases are the major retinal diseases caused by diabetes; unless an initial evaluation is conducted, these diseases always cause severe damage to the visual acuity, and it is irreversible [6, 38].

Growing life expectancy, hustling lifestyles, and other factors suggest that the number of people with diabetes is expected to increase [38]. For example, many patients with DED in China often overlook their situation and lack timely treatments leading to serious state development of DR [55]. Early intervention of abnormal signs prevents further deterioration of the condition and its effect on the impacted individuals and related medical costs. Therefore, the DED identification system enables either completely automate the eye-screening process or semi-automate eye-screening system. The first method requires a reasonable degree of accuracy, which is similar to that of the retinal experts. As per the British Diabetic Association (BDA) guidelines, a minimum level of 95% specificity and 80% sensitivity for sight-threatening DR detection must be obtained applied method [3]. Second option allows to downsize the large-scale mass-screening outputs to the potential DED cases, followed by human examination. Both scenarios significantly reduce the burden on skilled ophthalmologists and specialised facilities, making the process accessible to wider population, especially in low-resource settings.

After all, the application of deep learning to the clinical practice still has many challenges. An earlier paper dealt with ethical and political concerns in terms of database creation [1]. For this purpose, it has been difficult to obtain large-scale data on many diabetic eye diseases. Another challenge is that mild (early) classification problems consist of real clinical problems. Binary classification for diabetic eye disease prediction was the subject of previous studies. Even if Google has built a deep learning model that works better than ophthalmologists, their ’Inception-v3’ model was optimised for binary classification for DR identification based on the GoogLeNet structure Gulshan et al. [17]. This framework was evaluated after adding a wide image database gathered for only healthy and non-healthy DR screening from diabetes patients. For binary disease classification, Gulshan et al. stated a 93-96 percent recall but noted that recall is not enhanced while practicing with 60,000 image samples contrast with 120,000 image samples employing a private data.

Visual representations of the features acquired by CNNs demonstrate that the patterns being used for classification are a part of the image fully visible to the observer [53]. Moderate and severe class of the diabetic retinal images include macroscopic features on a scale designed for classification by current CNN architectures, such as those accessible from the ImageNet visual database. On the other hand, less than 1 % of the overall pixel volume, a degree of slight that is often difficult for human interpreters to identify, is the characteristics that differentiate mild from the normal disease.

This research indicates that mild-class DED classification should be established through further studies on automatic diagnosis using retinal fundus imaging. The first part of the experiment includes traditional image processing for enhancing mild DED features. Various conventional techniques for image processing have been implemented to extract DED lesions. Pre-trained CNN models using transfer learning provides excellent performance with object-oriented images such as flowers, cars, and animals but not efficient with lesion based medical images. So in this research we aim to objectify mild DED lesion via segmenting region of interest and transfer it to transfer learning and build CNN for further feature extraction. Following the elimination of the top layer (existing approach). A detailed review of 3 CNN architectures (including state-of-the-art architectures) was conducted. Secondly, the n layers were ’unfrozen’ and then re-trained to respond effectively to the details of the case-study of the application (proposed approach). Messidor, Messidor-2, retinal dataset were provided for system training. Two training sets were prepared with available dataset (i) before, and (ii) after preprocessing to measure potential accuracy improvement for Normal/Mild DED image classification.

As Mild DED tends to be incredibly difficult to discern from a normal retina due to few subtle indication of the impairment, an increase in the quality data was supposed to improve the visibility of pathological features. The top 1 CNN architectures with the top layer removed and re-trained were *VGG16*, yielding the accuracy of 83.43%, 89.13%, 88% for each (Table. 4). The lowest performance was obtained by *Xception* and *DenseNet21* respectively. The impact of fine tuning varied across the models. The observed improvement in accuracy was only minor, indicating the relative appropriateness of default pre-trained networks for DED classification tasks. In other terms, the CNN networks were able to identify Mild DED from a healthy retina despite having been trained on different images from the ImageNet repository. If no improvement in accuracy is obtained, the unfreezing is not advised result in unnecessary computational costs and time accrued. For build CNN model yielded the accuracy of 93.33%, 91.43%, 100% respectively.

For comparing the performance of the employed models, the 2 scenarios were considered, (1) before image preprocessing, and (2) after image preprocessing. In before preprocessing scenario, we trained our models with a raw dataset with data augmentation (geometric transformation) were applied to Messidor, Messidor-2, DRISTI-GS dataset, to avoid overfitting. In after image preprocessing scenario, dataset were preprocessed using various traditional image processing techniques which has increased the classification performance to 100% (the maximum accuracy achieved for Gl).

After evaluation of our high performed approach on Mild DR, Mild DME and Mild GL detection task, the maximum sensitivity of 100%, 94.44%, 100% and the maximum specificity of 86.67%, 88.24%, 100% were obtained. Thus, the early DED detection proved sufficient given the BDA standards, but still falling 9% and 6% short in terms of its specificity.

### Approach Limitations

Several deficiencies of the research have been established. First, Data set acquired for this experiment were obtained from publicly available which limits number of high quality mild DED images, only limited-to-moderate data set sizes were employed in the research. The approach also emphasises the value of an effective annotation process as having a direct effect on the output of the classifier. The Messidor, Messidor-2, retinal dataset has been validated and marked by professional ophthalmologists. Transfer Learning is used as a compensation procedure. Pre-trained CNN models in the wide-scale ImageNet database have been adopted in this study. To increase the size of the training sample set and to ease the data imbalance problem data were rotate, flipped, mirrored, etc. Second, the default model parameters were adopted for classification task (i.e. dropout, batch size, loss function, optimizer etc.). Finally, the ’black-box’ nature of Deep Learning-based solution is often criticized, causing resistance in the broader approach adopted by practitioners. However, with a build CNN using binary classifiers, we achieved state-of-the-art accuracy, the model performance degrades with the use of transfer learning. However, it is striving to ensure that more data might be more robust. Previous field research has confirmed that CNN’s ability to accommodate differences in size is limited and some have indicated that more data can not complement this inherent weakness in the case of retinal images [17].

## Conclusion

This paper proposes an approach that focuses specifically on the identification of mild DED among Normal instances as not adequately discussed in previous literature. According to the analytical aspect of Deep Learning, a variety of performance optimization techniques have been employed (1) Image enhancement, (2) feature enhancement, (3) data balance, and (4) fine-tune. The additional advantage of Deep Learning involves automatic recognition capabilities that are most selective between categories. Such an approach makes it possible to avoid technological constraints with the analytical, and sometimes subjective, approach of manual extraction of features. In addition, the analysis used composite data sets from various sources to determine the robustness of the system and its capacity to respond to real-world scenarios. The developed system enables the standardisation of labour-intensive eye-screening processes and satisfies as an auxiliary diagnosing reference, while avoiding human subjectivity.

## Data Availability

Public dataset

## Footnotes

1 https://github.com/yiweichen04/retina_dataset/tree/master/dataset.
